# Thermal and Optical Characterization of Polycarbonate Reflectors Doped with Titanium Dioxide Using Thermography

**DOI:** 10.3390/ma18071628

**Published:** 2025-04-02

**Authors:** Isabella Luísa Vieira Aquino Cassimiro, Juan Ignacio Tomsich, Matheus Pereira Porto, Rosemary do Bom Conselho Sales, Izabella Helena Werneck Soares Rezende, Nathan Funchal de Rezende, Maria Teresa Paulino Aguilar

**Affiliations:** 1 Graduate Program in Mechanical Engineering, Federal University of Minas Gerais (UFMG), Belo Horizonte 31270-901, MG, Brazil; matheusporto@ufmg.br (M.P.P.); izahwsrezende@gmail.com (I.H.W.S.R.); nathanfr13@gmail.com (N.F.d.R.); teresa@ufmg.br (M.T.P.A.); 2 Graduate Program in Engineering, Universidad Tecnologica Nacional Facultad Regional Haedo, Haedo B1706, Argentina; jtomsich576@alumnos.frh.utn.edu.ar; 3 Graduate Program in Design, State University of Minas Gerais (UEMG), Belo Horizonte 30160-042, MG, Brazil; rosemary.sales@uemg.br

**Keywords:** polycarbonate, automotive reflector, metallization, TiO_2_ nanoparticles, infrared thermography, lighting performance, thermal diffusivity

## Abstract

Automotive reflectors used in headlamps and rear lamps are typically made of polycarbonate. However, this polymer has low light reflectivity. To enhance its reflective properties, it undergoes a metallization process, which significantly increases production costs. Therefore, it is of interest to develop polymers that do not require metallization for the manufacturing of automotive reflectors. In this regard, the use of polycarbonate reinforced with titanium dioxide nanoparticles may be an alternative. Studies indicate that incorporating these nanoparticles can improve the degradation temperature and mechanical properties of the composites. In the case of automotive reflectors, in addition to degradation due to temperature, it is crucial to assess the thermal diffusivity and reflectivity of these composites, thus ensuring the lighting performance of the component. Studies on such characteristics in polycarbonates with titanium dioxide nanoparticles are mostly limited to investigations of hardness and optical properties using Raman and UV–Vis spectroscopy tests. This article investigates the thermal and lighting performance of polycarbonate (PC) samples with 10 wt% titanium dioxide (TiO_2_) nanoparticles and automotive reflectors with the same chemical composition. The thermal stability of PC and PC-10%TiO_2_ was analyzed by thermogravimetry (TGA), whereas the reflectors were evaluated using active infrared thermography. Spectral thermographic analysis in the mid- and long-wave infrared range provided thermal diffusivity data for the polycarbonates and offered important insights into their optical behavior under operational conditions (up to 70 °C). Furthermore, illumination tests were conducted on PC-10%TiO_2_, using metalized polymeric reflectors commonly employed in the automotive industry as a reference. The TGA results showed that incorporating 10 wt% TiO_2_ into PC increased the degradation temperature from 167 °C to 495 °C. The long-wave infrared emissivity of PC-10%TiO_2_ (averaging 0.96) was 3% lower than that of polycarbonate. PC-10%TiO_2_ exhibited a thermal diffusivity of 0.20 mm^2^/s, which was 28.6% lower than that of PC, indicating greater thermal inertia due to the presence of nanoparticles. The lighting performance of the PC-10%TiO_2_ reflector was on average 4% lower than that of a commercially available metallized polycarbonate reflector. However, for automotive reflectors, this value meets the sector’s regulatory criteria. These findings suggest that PC-10%TiO_2_ has potential for use in the production of internal vehicle lighting reflectors, without significantly compromising light reflectivity, while offering the advantages of thermal stability and reduced heating around the reflector.

## 1. Introduction

In the automotive sector, both internal and external lamps are essential components, as they contribute to vehicle signaling, safety, comfort, and esthetics [1]. Lamps comprise the lens, the reflector, and the light source. Reflectors play a key role in directing and reflecting light. The most used material for manufacturing reflectors is polycarbonate. Although polycarbonate (PC) possesses suitable thermal, mechanical, and impact resistance properties, it has low light reflectivity [2,3,4,5,6,7]. To overcome this drawback, a metallization process is applied to the reflectors. Metallization involves the deposition of a thin aluminum layer onto the polymer surface through evaporation [3,8,9]. This process is not only costly but also time-consuming, particularly when dealing with complex geometries [10,11,12]. A cost- and time-effective alternative to eliminate the metallization stage is the incorporation of titanium dioxide nanoparticles into PC, as this compound has been extensively studied for various applications due to its optical properties [13].

Literature studies using PC films with low TiO_2_ content (up to 6 wt%) indicate that increasing the TiO_2_ proportion enhances the composite’s mechanical and/or thermal properties, but the optical band gap values reduce with an increase in the TiO_2_ content of the nanocomposites [14,15,16,17,18,19,20,21,22,23,24]. Regarding thermal performance, most of these studies assess stability and degradation temperature as a function of TiO_2_ content. However, thermal conductivity and/or diffusivity, as well as heat reflection and lighting performance, have not been evaluated. Studies on PC with TiO_2_ concentrations above 6 wt%, whether related to thermal or optical performance, remain scarce. Jawad and Ahmed [25] investigated the effects of incorporating titanium dioxide nanoparticles in proportions ranging from 2% to 10% in PC/wood fiber composites. They assessed the material’s morphological, mechanical, thermal, electrical, and intrinsic properties. The results showed that adding TiO_2_ nanoparticles increased the thermal conductivity of the composites, with the highest values observed at around 3 wt% TiO_2_. Eskandari [26] conducted optical property studies using PC with 5% and 10% TiO_2_, employing Raman spectroscopy tests and UV–Vis spectroscopy, and showed that, by increasing the percentage of nanoparticles to PC, the band gap of the composites started to decrease, and the hardness values increased.

In addition to luminous performance, automotive light reflectors must also exhibit thermal stability to maintain their efficiency over time. They should have high reflectivity in both the visible and infrared spectra, along with low thermal diffusivity. High reflectivity in the long-wave infrared spectrum minimizes heat absorption, preventing performance degradation or damage to adjacent components. Low thermal diffusivity in the material hinders rapid heat propagation through the reflector, avoiding excessive heating of the components near the light source. Both properties are crucial to ensure optimal functionality of the lighting system. Despite the well-established knowledge of the thermal and optical properties of polycarbonate [27,28] and TiO_2_ [29,30,31], there is a lack of information regarding the thermal diffusivity and heat reflection of TiO_2_-modified PC composites. Moreover, no data have been found on the lighting performance of PC with 10 wt% TiO_2_ (PC-10%TiO_2_). Thus, a knowledge gap exists regarding the contribution of high TiO_2_ concentrations to the thermal stability, reflectivity, and thermal diffusivity of PC-10%TiO_2_. In this context, to further advance the field, this study investigates the optical and thermal performance of the raw material and reflectors made from PC with 10 wt% TiO_2_. Thermogravimetric analysis, active infrared thermography, and lighting performance testing were employed in this study.

Active infrared thermography, using a lamp and a climate chamber as the heat source, was chosen because it had been previously used to evaluate the thermal properties of components as a whole [32,33,34,35,36]. However, to achieve so, it is necessary to know the emissivity of the materials precisely. Emissivity is the capacity of a surface to emit thermal radiation when compared to that of a black body. This factor depends on the wavelength, the direction of observation in relation to the surface, and the temperature of the material [27]. The determination of emissivity by infrared thermography is commonly performed by the “tape method” [37]. However, this method can be improved by combining it with the two-color method to determine the emissivity of PC-10%TiO_2_, using two thermal cameras that operate in different regions of the infrared spectrum, thus adopting a spectral approach [23,38,39,40]. The combined use of the tape method with the two-color method as a technique for determining optical properties at temperatures close to room temperature represents, to the best of the authors’ knowledge, an unprecedented contribution to the field of materials. In this sense, it is believed that the methods discussed here can support the use of thermography in other studies.

## 2. Materials and Methods

### 2.1. Materials

**Figure 1:**
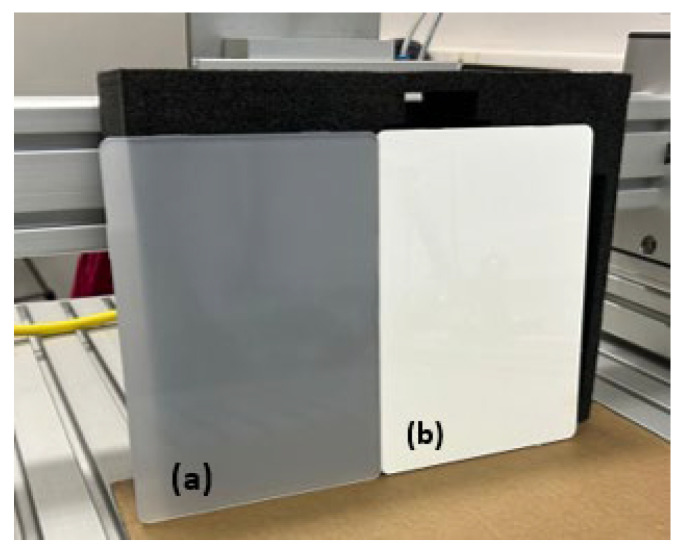
(**a**) Polycarbonate plates and (**b**) polycarbonate plates with 10% of TiO_2_.

**Figure 2:**
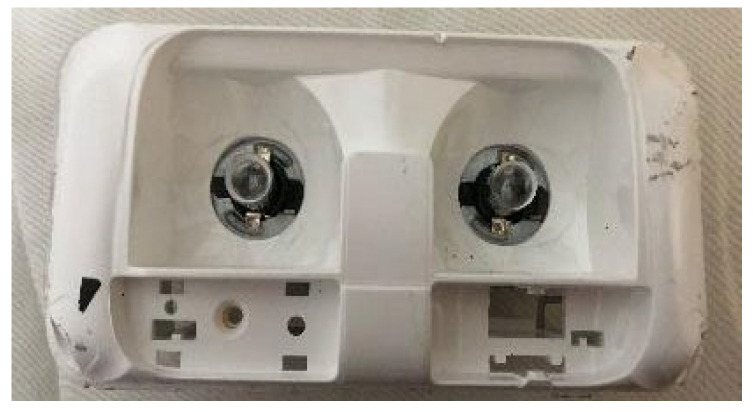
Polycarbonate reflector with the addition of 10% titanium dioxide (PC-10%TiO_2_).

Samples were analyzed, fabricated from PC plates with and without the incorporation of 10 wt% TiO_2_ nanoparticles (Figure 1). For thermal performance and luminosity tests, PC reflectors with the addition of 10 wt% titanium dioxide were specifically produced for the experiment (Figure 2). The reflectors were manufactured with dimensions of 136.7 mm in length, 70.7 mm in width, and 48.1 mm in height. Both the plates and the reflectors were custom-made for this study and processed under the supervision of the automotive manufacturer Stellantis, Betim, MG, Brazil.

### 2.2. Methods

The samples were prepared in the shapes and sizes corresponding to each type of test. TGAs were performed on the samples in powder form, while thermographic tests were conducted on the samples in the form of disks. Both sample types were taken from PC plates with and without the addition of 10% TiO_2_. PC-10%TiO_2_ reflectors were subjected to thermal performance and lighting tests.

#### 2.2.1. Determination of the Thermal Stability of the Materials

The thermal stability of PC with and without the addition of 10%TiO_2_ was evaluated through thermogravimetry using 10 g of powder from each material. The TA Q600 model equipment (TA Instruments, New Castle, DE, USA) was used, within a temperature range of 40 °C to 800 °C, with a heating rate of 20 °C/min in an inert nitrogen dioxide (NO_2_) atmosphere.

#### 2.2.2. Determination of the Emissivity of the Materials

**Figure 3:**
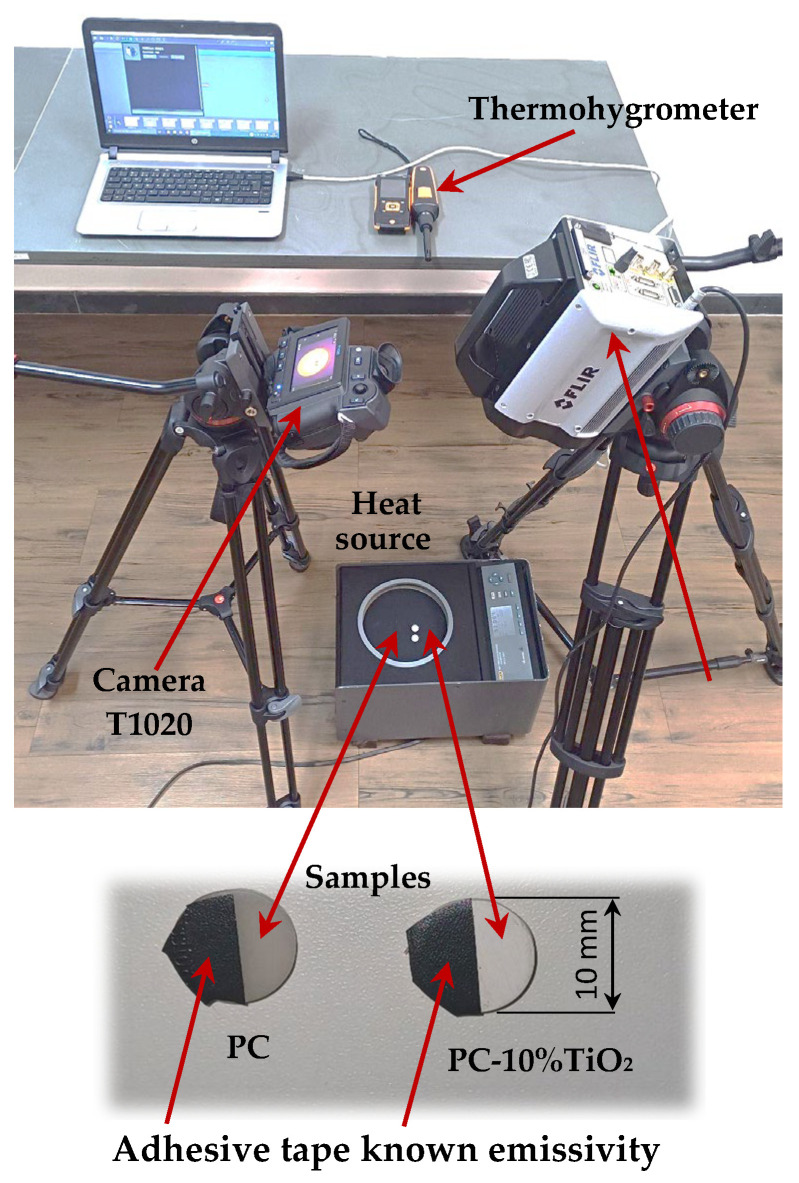
Experimental setup for emissivity measurement.

Firstly, quantitative active infrared thermography was used to calculate the emissivity of the materials [32,33,34,35,36]. The emissivity of PC, with and without the addition of 10% TiO_2_, was determined using the “tape method”. The samples were cut from the plates in the form of disks with a diameter of 10 mm and a thickness of 3.5 mm [40]. The test involved using an adhesive tape (Scotch™ (St. Paul, MI, USA) by 3M) with a known emissivity [37] for comparative measurements. The tape was applied to half of the sample surfaces, as shown in Figure 3. The samples were then placed on a heated surface. After the necessary time for the system to reach thermal equilibrium, thermograms were recorded by the thermal camera. For measurements using the two-color method, two cameras operating in distinct spectra were employed [38]. Four measurement points were recorded with both thermal cameras: two on the tapes and two on the surface of the materials. The cameras used were the Flir T1020 (FLIR Systems, Wilsonville, OR, USA) (8 μm to 14 μm) and the Flir X6801sc (3 μm to 5 μm). The experimental setup is shown in Figure 3. Humidity and ambient temperature were kept under control, monitored by a thermohygrometer Testo 605i (Testo SE & Co. KGaA, Lenzkirch, Germany).

The emissivity of the tape was considered known, which allowed the use of Equation (1) to obtain the temperature of the disks. Considering thermal equilibrium between the regions of the sample with and without the tape, the temperature of the taped area could be assumed to be equal to that of the untaped area, leaving the emissivity as the one unknown in Equation (1) for the untaped region, as shown in Table 1. Since this was a gray surface, the emissivity measured in both spectra was expected to be similar.

(1) $$L_{\lambda ,abs\;}=\int_{\lambda }^{0}\left[L_{\lambda \;}\left(T_{ob}\right)\varepsilon_{\lambda ,ob}\left(1-\varepsilon_{\lambda ,atm}\right)\tau_{lens,\lambda }R_{\lambda }+L_{\lambda \;}\left(T_{refl}\right)\left(1-\varepsilon_{\lambda ,ob}\right)\tau_{lens,\lambda }R_{\lambda }+L_{\lambda \;}\left(T_{atm}\right)\varepsilon_{atm,\lambda }\tau_{lens,\lambda }R_{\lambda }\right]d\lambda \;$$

In Equation (1), *λ* represents the wavelength (µm), *L_λ,abs_* is the radiance (W/m^2^) calculated for *λ*, *L_λ_* is the radiance (W/m^2^) detected by the camera at *λ*, *ε_λ,atm_* and *ε_λ,ob_* are the emissivity of the atmosphere and the object (unknown), respectively, and *T_ob,_ T_refl_*, and *T_atm_* are the temperatures of the object (K), the reflected surface, and the atmosphere, respectively. The transmissivity of the thermal lens *τ_lensλ_* (dimensionless) and the spectral responsivity of the detector *R_λ_* (dimensionless) were obtained from the manufacturer’s database.

#### 2.2.3. Determination of the Thermal Diffusivity of the Material

**Figure 4:**
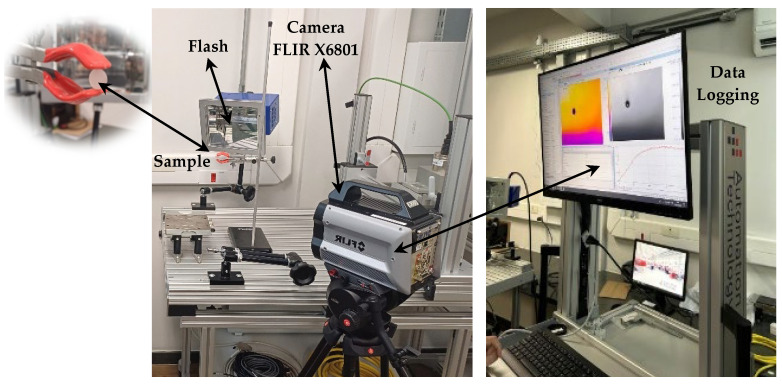
Experimental measurement setup of thermal diffusivity.

Active infrared thermography was also used to measure and quantitatively determine the thermal diffusivity of the materials. The measurement was based on ASTM E1461-13—thermal diffusivity by the flash method—[41] which considers the use of a correction factor to calculate the average thermal diffusivity of the material. The method evaluates the thermal diffusivity of homogeneous solid materials that are opaque to the applied radiation [41]. Samples in the form of disks with a diameter of 10 mm and a thickness of 3.5 mm were used, having an area smaller than that of the energy beam. The Flir X6801sc (FLIR Systems, Wilsonville, OR, USA) thermographic camera with spectral sensitivity between 3 μm and 5 μm, a support to position the samples, a lamp (6 kJ linear) to generate the flash, temperature detectors, and equipment for data recording were used (Figure 4).

Thermal diffusivity was calculated from measurements at seven points on each sample and as a function of the time it took for the heat to propagate from the front surface of the samples to the back surface. The test sequence consisted of determining the temperature of the sample over time. Immediately after the application of the flash, the camera captured the radiation on the opposite side of the incidence for 30 s. Ten temperature measurements (*T*) were recorded every second. Using the data obtained over the 30 s, a *T* curve as a function of time (*t*) was plotted. In this way, the time to reach the temperature after 30 s could be calculated, considering the initial temperature of the sample. With this value, the thermal diffusivity (*α*) could be calculated using Equation (2). ASTM E1461-13 [41] recommends using three-time measurements, *t_0.25_, t_0.50_, and t_0.75_*, which represent the times to reach 25%, 50%, and 75% of the maximum temperature.

(2) $$\alpha ={\;k}_{x}\;l^{2}\;/\;t_{x}$$

where *α* is the thermal diffusivity (m^2^ s^−1^), *l* is the sample thickness (mm), ${\;k}_{x}$ is a constant related to the speed to reach the temperature in time $t_{x}$, and $t_{x}$ is the time to reach 25%, 50%, and 75% of the maximum temperature (s).

To calculate the representative thermal diffusivity of the material, it was necessary to use the ${\;k}_{r}$ factor calculated by Equation (3). If the ratio of t_0.75_/t_0.25_ (s) was close to 2.272, as stipulated by ASTM E1461-13 [41], the corrected thermal diffusivity was recalculated (Equation (4)).

(3) $${\;k}_{r}=-0.3461467+0.361578\;\left(\frac{t_{0.75}}{t_{0.25}}\right)$$

where *t*_0.25_ and *t*_0.75_ (s) correspond to the time to reach 25% and 75% of the maximum temperature.

(4) $$\alpha_{C}=\alpha_{0.5}\;.\frac{{\;k}_{r}}{13885}$$

where $\alpha_{C}$ is the corrected thermal diffusivity, and $\alpha_{0.5}$ is the thermal diffusivity for the time corresponding to 50% of the time taken to reach the maximum temperature.

#### 2.2.4. Determination of the Thermal Performance of the Reflector

For this study, a front roof lamp from one of Stellantis’ vehicles was used. The thermal performance was evaluated because, during operation, the reflector is positioned very close to the lamps within the lighting unit. The PC-10%TiO_2_ reflectors were manufactured by Olsa of Brazil, following the company’s compliance standards.

**Figure 5:**
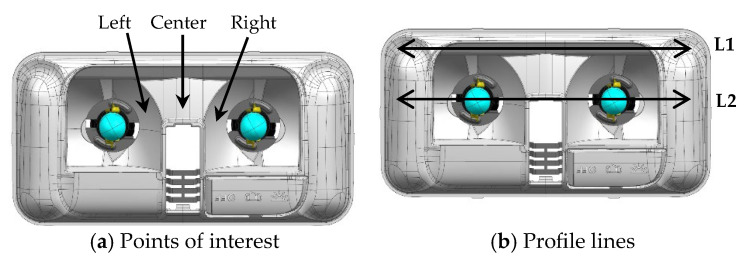
Points of interest for thermographic reading and profile lines on the reflector PC-10%TiO_2_: (**a**) points of interest and (**b**) profile lines.

Active infrared thermography was used to estimate the temperatures during the heating of the reflector. A Flir ThermoCAM T420 thermal camera was employed, along with a Fuji N480D (Fujifilm Corporation, Tokyo, Japan) climate chamber, to maintain the reflector’s temperature, simulating vehicle operation. The tests followed a protocol specifically developed for this research. The thermal camera was placed one meter away from the reflector, which was positioned inside the climate chamber, regulated at a temperature of 60 °C. The lamps were connected to a 12 V electrical current and remained switched on for 60 min, the duration recommended by the manufacturer to operate the lamp without softening the material. After this period, both the lamps and the climate chamber were switched off, and temperature measurements were taken. The experimental procedure was conducted both qualitatively and quantitatively. The FLIR Research Studio software (2024.5.0) was used to extract temperature values from three regions of interest: two points at the sides of the lamps and one at the midpoint between them (Figure 5a). Additionally, two profile lines were drawn across each sample: one (L1) passing through a region where the heat from the lamps was not expected to have a direct influence and another (L2) crossing both lamps (Figure 5b).

The thermal camera was configured to obtain apparent temperature values, considering a reflected temperature of 20 °C, an atmospheric temperature of 20 °C, a relative humidity of 50%, and an emissivity of 0.95. Since it was a gray-diffuse surface, Equation (5) was used to estimate the actual temperature of the materials *T_obj_* (K), where *T_full_* represented the apparent surface temperature, *T_refl_* and *T_atm_* corresponded to the reflected and atmospheric temperatures, respectively, both assumed to be 20 °C, and the transmissivity *τ_atm_* = 1 (dimensionless). The measurement uncertainty provided by the manufacturer was ±2%.

(5) $$T_{full}={\left[{\varepsilon \tau }_{atm}T_{obj}^{4}+\left(1-\varepsilon \right)\;\tau_{atm}T_{refl}^{4}+\left(1-\tau_{atm}\right)\;T_{atm}^{4}\right]}^{0.25}$$

#### 2.2.5. Determination of the Lighting Performance

The illuminance level was measured in lux, comparing the PC-10%TiO_2_ reflector with a metalized polycarbonate reflector used in Stellantis’ assembly line. The analysis considered the regions defined by the manufacturer’s internal lighting level standards. The reflectors were installed in the front roof section of a vehicle provided by the manufacturer. Nine points of interest were defined inside the vehicle for measurement: one point in the central console (Region 1), three points on the seat/backrest of the driver’s and passenger’s seats (Regions 2 to 8), and one point on the side of each front door (Regions 5 and 9). The reflection range used was the one required by Stellantis for lights installed in these regions of the vehicle, as shown in Table 2. The Konica Minolta CL-200 lux meter (Konica Minolta, Inc., Tokyo, Japan) was used, and a dark chamber was employed to eliminate any external light interference.

## 3. Results and Discussion

### 3.1. Thermal Stability of Materials

**Figure 6:**
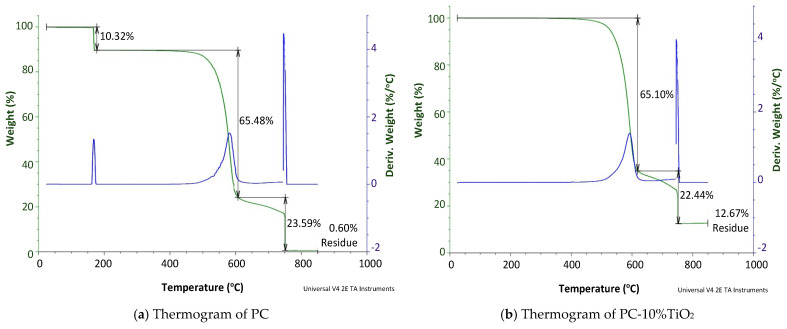
Thermogravimetric curve of PC-10%TiO_2_: (**a**) thermogram of PC and (**b**) thermogram of PC-10%TiO_2_.

The thermogravimetric analysis curves for PC and PC-10%TiO_2_ are presented in Figure 6. For PC, an initial weight loss of 10.32% was observed at 167 °C due to the presence of residual solvent or monomer [42]. After this loss, the material remained stable up to 469 °C, when a continuous mass loss began in two stages, with losses of 65.48% and 23.59% at temperatures of 602 °C and 752 °C, respectively. No further weight loss was apparently observed beyond 698 °C, leaving a residue of 0.60%. The degradation mechanisms involved in each stage were complex, and there is no consensus in the literature regarding the chemical nature of the reactions involved [43]. Similar results are reported in the literature [44].

When analyzing the results for PC-10TiO_2_, it should be considered that TiO_2_ has thermal stability up to 1200 °C; therefore, within the temperature range considered for the experiments, no mass loss related to this compound was observed [26,29,30]. In PC-10%TiO_2_, the material was thermally stable up to 495 °C, with no initial loss around 167 °C, as seen in PC. This was likely due to TiO_2_ nanoparticles acting as a physical barrier, hindering the volatilization of residues and/or solvent. Two stages of continuous mass loss were also observed, with losses of 65.10% and 22.44% at temperatures of 609 °C and 748 °C, respectively, probably associated with the PC polymer. Mass stabilization for PC-10%TiO_2_ occurred around 749 °C, with a residue of 12.67%. Considering that the residue for pure PC was 0.6%, the data for PC-10%TiO_2_ indicated that effective incorporation of the oxide occurred [20,45]. Regarding the mass loss rate, the temperature at which the highest rate of weight loss occurred was either reduced or remained unchanged. The higher thermal stability, up to 495 °C, made the material more suitable for use in automotive reflectors, which can heat up to around 200 °C due to the lamp.

### 3.2. Emissivity of the Materials

**Figure 7:**
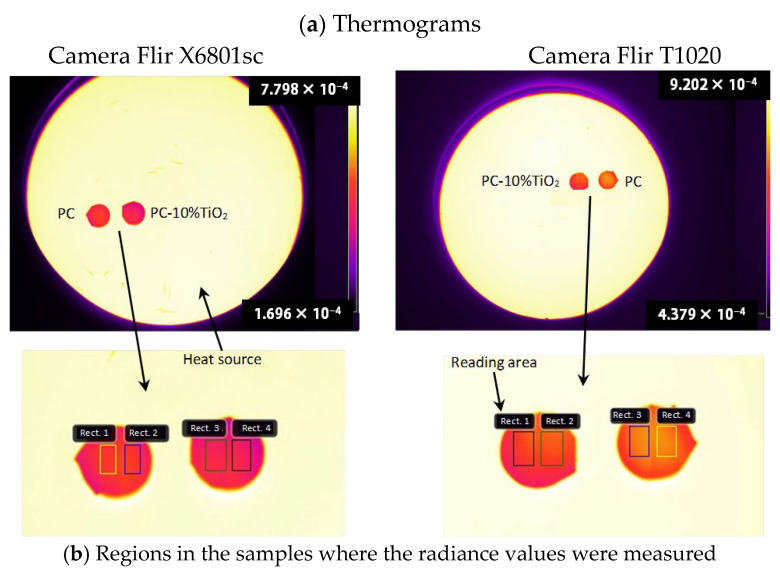
Radiance data obtained with the thermal cameras: (**a**) thermograms and (**b**) regions in the samples where the radiance values were measured.

In Figure 7a, the radiance thermograms for the Flir X6801sc and T1020 camera are shown, and Figure 7b shows the regions in the samples where the radiance values were measured for each camera. From the radiance values, it was possible to calculate the emissivity and the reflected surface temperature of each sample for each camera used, applying Equation (1).

The results obtained are presented in Table 3. The small difference between the emissivity results obtained from the different thermal cameras demonstrated that the emissivity could indeed be considered as a gray surface [23,39,40]. However, the comparison between the materials showed that the emissivity of the PC-10%TiO_2_ material was lower in both measurements. This indicated that this material had higher reflectivity and, consequently, minimized the transfer of heat by radiation [20,46]. Materials with low emissivity tend to degrade less over time because they absorb less heat, which could cause expansion and contraction, leading to wear. Therefore, the reflectors made with PC-10%TiO_2_ would have a more favorable thermal behavior over their lifespan.

### 3.3. Thermal Diffusivity of the Material

**Figure 8:**
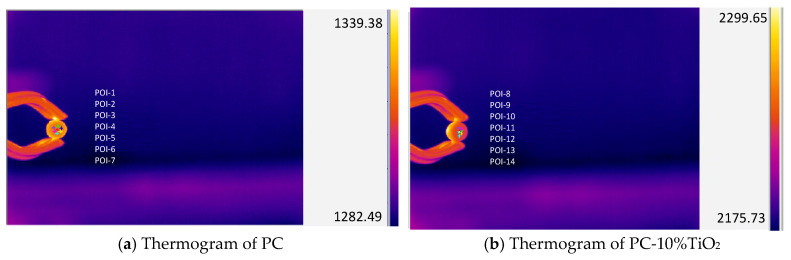
Thermograms for determining the thermal diffusivity as a function of time: (**a**) thermogram of PC and (**b**) thermogram of PC-10%TiO_2_.

**Figure 9:**
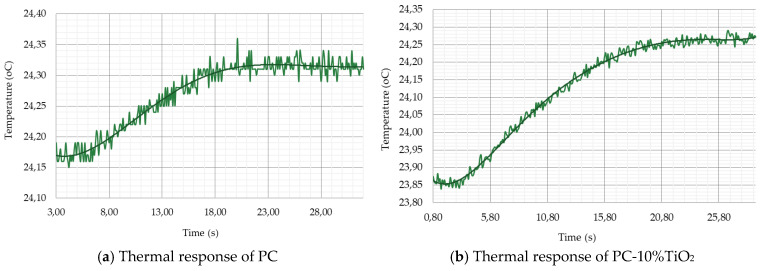
Thermal response of samples: (**a**) thermal response of PC and (**b**) thermal response of PC-10%TiO_2_.

The thermograms of PC and PC-10%TiO_2_ (Figure 8) showed the seven points (seven different points inside the samples) where each frame of the measurement sequence was obtained. From these measurements, an average temperature value for the PC and PC-10%TiO_2_ was calculated, and this value was chosen as representative of the temperature of the entire surface at a given time. Using the average values obtained over 30 s, the temperature curve was plotted as a function of time. As a result, the temperature values for points (t_0.25_, *t*_0.50_, and *t*_0.75_) were determined, representing 25%, 50%, and 75% of the temperatures reached. A polynomial fitting curve was plotted to facilitate the visualization of the temporal evolution of the thermal response (Figure 9). It was observed that PC increased its temperature by 0.4 °C in 30 s. When using PC-10%TiO_2_, the temperature increased by only 0.25 °C over the same period, indicating the greater influence of the addition on the thermal behavior of the PC, which thus heated less, effectively maintaining the internal temperature of the environment in which it had been placed.

Table 4 presents the results obtained that allowed for the calculation of the corrected thermal diffusivity. The average corrected thermal diffusivity value for the PC sample was 0.28 mm^2^/s. For the PC-10%TiO_2_ sample, it was 0.20 mm^2^/s. The PC-10%TiO_2_ exhibited a lower thermal diffusivity coefficient, indicating greater thermal inertia. This was due to the increase in the TiO_2_ nanoparticle content, which promoted the formation of a more complex network which hindered heat dissipation [47,48]. Therefore, heat was not quickly dissipated into the environment, potentially causing overheating and degradation of the material. As a result, the material was more capable of maintaining a stable internal temperature. As an advantage, the reflector had less interference with the surrounding temperature.

### 3.4. Thermal Performance of the Reflector

**Figure 10:**
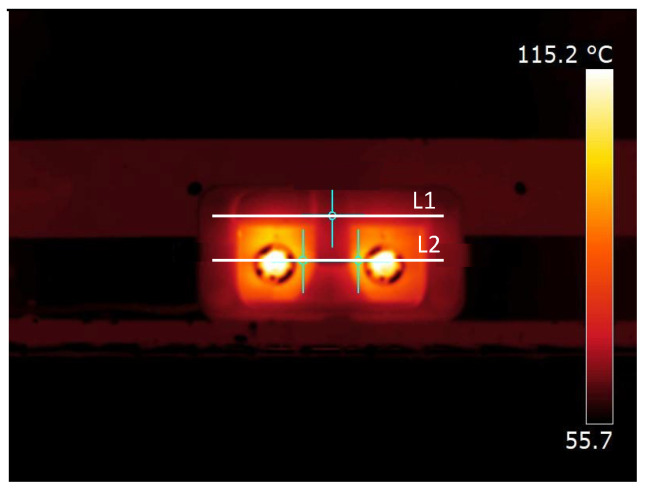
Thermogram of the three points of interest and profile lines of the reflector PC-10%TiO_2_.

The thermogram in Figure 10 shows the reflector PC-10%TiO_2_/light with the three points of interest and the profile lines, with L1 passing outside the region of the lamps and L2 passing through the concave part.

**Figure 11:**
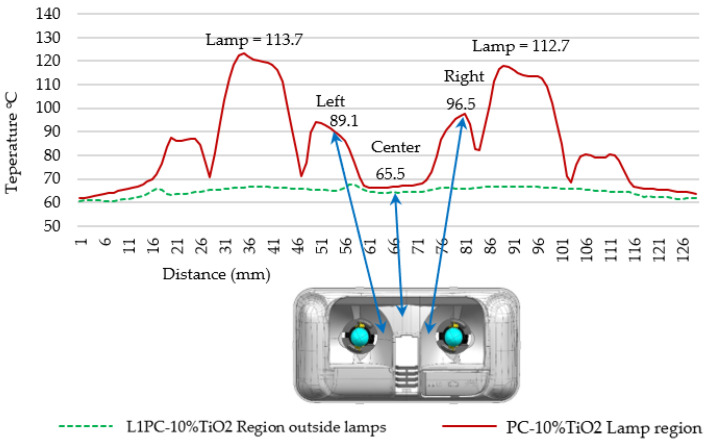
Values of apparent temperatures along lines L1 and L2 of the structure of the PC-10%TiO_2_.

Figure 11 shows the apparent temperatures along the two lines (L1 and L2). The temperatures in L1 were close to the temperature of the climate chamber, around 65 °C, indicating that the PC-10%TiO_2_ was slightly affected by the heat from the lamps in that region. In line L2, the apparent temperature of the PC-10%TiO_2_/light reflector was 89.1 °C on the left and 96.5 °C on the right. In the central region, the apparent temperature was significantly lower, reaching 65.5 °C on the reflector body, very close to the temperatures of L1, meaning that it was only slightly affected by the heat from the lamps.

As it was a gray-diffuse surface, Equation (5) was used to estimate the temperature reached by the PC-10%TiO_2_ structure, as shown in Table 5 and Figure 11. The thermal radiation components from the lamps reflected over time on the concave surfaces, contributing to the considerable difference between the apparent and real temperatures. In this case, the lamp glass was considered opaque in the LWIR (long-wave infrared), leading to a reflected temperature (*T*_refl_) of 113.7 °C on the left side and 112.7 °C on the right side. The emissivity of PC-10%TiO_2_ was expected to decrease with the emission angle [49], meaning that the temperature should have slightly decreased at the extremes of the concave surface, which can be observed upon closer inspection of the thermography analysis in Figure 11. The surface of PC-10%TiO_2_ reached less than 100 °C, staying below the material’s degradation temperature, as shown in Figure 6.

### 3.5. Lighting Performance

Table 6 presents the results obtained during the illumination level test of lamps manufactured with a PC-10%TiO_2_-based reflector and a metalized polycarbonate reflector (PC + Met). A variation of 7.1% to 0.9% was observed between the measured values at the nine points when using each type of lamp.

Overall, the reflective efficiency of PC-10%TiO_2_ was 4% lower than that of the metalized reflector. This was likely due to the glossy surface of PC + Met, which enhanced reflection. In contrast, the PC-10%TiO_2_ reflector exhibited lower light dispersion due to the presence of TiO_2_ nanoparticles, which reduced surface gloss [50,51].

Despite the slightly lower values, the illumination level of the PC-10%TiO_2_ reflector remained within the light reflection range required by Stellantis for lights installed in the upper front region of the vehicle.

## 4. Conclusions

A thermogravimetric analysis revealed that PC-10%TiO_2_ exhibited thermal stability up to 495 °C, significantly higher than PC, which showed an initial mass loss of 10.32% at around 167 °C. Notably, both materials displayed peak mass losses at similar temperatures, approximately 65% and 23%, respectively, values which aligned with the decomposition data of PC, as TiO_2_ remained stable up to 1200 °C. The residual mass data confirmed the effective incorporation of TiO_2_ nanoparticles into the PC matrix.

The emissivity measurements from thermal cameras indicated gray-surface behavior for both materials. PC-10%TiO_2_ demonstrated a lower emissivity than PC, suggesting enhanced reflectivity and reduced radiative heat transfer, which could contribute to improved long-term durability. These properties make it a promising candidate for reflector applications.

Furthermore, PC-10%TiO_2_ exhibited a lower thermal diffusivity coefficient compared to PC, suggesting that the nanoparticles impeded heat dissipation, potentially leading to increased material temperatures. When the PC-10%TiO_2_ reflector was integrated into the interior dome lamp and operated for 60 min, it reached a maximum temperature of approximately 91 °C, representing about 20% of its degradation temperature.

Regarding lighting performance, the PC-10%TiO_2_ reflector showed an average reflection variation of 4% compared to a metallized reflector, yet this value remained within the manufacturer’s acceptable range for vehicle roof interior lighting.

These results demonstrate that PC-10%TiO_2_ is a viable alternative for reflector production. It maintains acceptable light reflection while offering the benefit of reduced heat transfer to surrounding areas.

Future research should explore the broader applications of these findings, particularly within the context of the circular economy and other relevant industrial sectors.

## Figures and Tables

**Table 1:** Plots that make up the radiation signal in the thermographic measurement.

Sample	Target		Atmosphere		Thermal Camera Image (Filters, Lenses, and Sensors)		Full Simulation
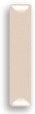			$L_{\lambda \;}\left(T_{ref}\right)\left(1-\varepsilon_{\lambda ,ob}\right)\left(1-\varepsilon_{\lambda ,atm}\right)$		$L_{\lambda \;}\left(T_{refl}\right)\left(1-\varepsilon_{\lambda ,ob}\right)\left(1-\varepsilon_{\lambda ,atm}\right)\tau_{lens,\lambda }R_{\lambda }$		
	$L_{\lambda \;}\left(T_{ref}\right)\left(1-\varepsilon_{\lambda ,ob}\right)$		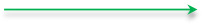		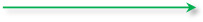		$L_{\lambda ,abs\;}$
	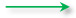		$L_{\lambda \;}\left(T_{ob}\right)\varepsilon_{\lambda ,ob}\left(1-\varepsilon_{\lambda ,atm}\right)$		$L_{\lambda \;}\left(T_{ob}\right)\varepsilon_{\lambda ,ob}\left(1-\varepsilon_{\lambda ,atm}\right)\tau_{lens,\lambda }R_{\lambda }$		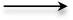
			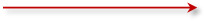		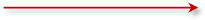		
	$L_{\lambda \;}\left(T_{ob}\right)\varepsilon_{\lambda ,ob}$		$L_{\lambda \;}\left(T_{atm}\right)\varepsilon_{atm,\lambda }$		$L_{\lambda \;}\left(T_{atm}\right)\varepsilon_{atm,\lambda }$		
	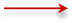		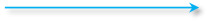		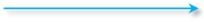		
		Environmental Component		Target		Atmosphere	

Source: Adapted from Flir System.

**Table 2:** Luminosity values in lux required.

Location by Region	Points Inside the Vehicle	Measured Points	Manufacturer’s Reflection Range (lux)
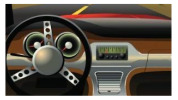	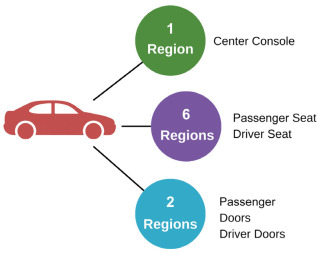	1	˃4 ˂40
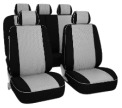		2–3–4 6–7–8	˃15 ˂40
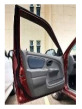		5–9	˃4 ˂40

**Table 3:** Values obtained for temperature and emissivity.

Sample	Calculated Temperature (°C)		Emissivity	
	Camera X6801sc	Camera T1020	Camera X6801sc	Camera T1020
PC	62.30	67.80	0.96	0.95
PC-10%TiO_2_	59.60	65.20	0.93	0.92

**Table 4:** Results of the determination of thermal diffusivity.

Material	Curve Points	*k_x_*	Temperature	Thermal Diffusivity (mm^2^/s)	*k_r_*	Corrected Mean Thermal Diffusivity (mm^2^/s)
PC	25%	0.09272	24.20	0.28	0.14	0.28
	50%	0.13879	24.24	0.29		
	75%	0.21049	24.27	0.29		
PC-10%TiO_2_	25%	0.09272	23.96	0.18	0.15	0.20
	50%	0.13879	24.06	0.26		
	75%	0.21049	24.16	0.23		

**Table 5:** Apparent and actual temperature for the PC-10%TiO_2_ reflectors.

Sample	Output	Temperature (°C)		
		Left	Center	Right
PC-10%TiO_2_	Apparent temperature	89.1	65.5	96.5
	Actual temperature	78.1 (*ε* = 0.8, *Τ_refl_* = 113.7 °C)	67.2 (*ε* = 0.9, *Τ*_refl_ = 30 °C)	90.9 (ε = 0.2, *Τ*_refl_ = 112.7 °C)

**Table 6:** Reflector lighting performance.

Measured Points	Manufacturer’s Lighting Limits Min/Max. (lux)	Reflector PC + Met (lux)	Reflector PC-10%TiO_2_ (lux)	Variation (%)
1	>4 <40	21.1	19.6	−7.1
2	>15 <40	21.1	20.6	−2.4
3	>15 <40	21.0	19.9	−5.2
4	>15 <40	17.6	16.7	−5.1
5	>4 <40	19.2	18.5	−3.6
6	>15 <40	17.2	16.7	−2.9
7	>15 <40	21.0	19.6	−6.7
8	>15 <40	21.1	20.9	−0.9
9	>4 <40	19.3	18.6	−3.6

## Data Availability

The original contributions presented in this study are included in the article. Further inquiries can be directed to the corresponding author.
